# Doing Well and Doing Good: Achievement and Social Life Goals of Gifted Korean Youths

**DOI:** 10.3390/bs16091539

**Published:** 2026-09-01

**Authors:** Jenny Yang, Seokhee Cho

**Affiliations:** Department of Administrative and Instructional Leadership, St. John’s University, Queens, NY 11439, USA

**Keywords:** gifted adolescents, Korean gifted education, life goals, achievement goals, social goals, family processes, paternal involvement

## Abstract

This study examined life-goal profiles among Korean gifted adolescents, focusing on two domains: achievement/career-oriented goals and social/relationship-oriented goals. Rather than assuming that gifted students are uniformly performance-driven, the study explored whether students who strongly endorsed both goal domains (dual-goal orientation) differed from students who reported comparatively lower endorsement of both domains (low-goal orientation). Independent-samples *t*-tests results indicated that the students from the two goal profile groups differed across several domains of academic and psychosocial experience. The dual-goal students reported significantly higher middle-school GPA, more supportive family environment, and higher paternal participation than low-goal students. They also perceived more advantages of being identified as gifted such as greater access to diverse opportunities. The groups did not differ, however, in study-related stress. Supplementary bivariate regression results show that the dual-goal profile was most clearly distinguished by family support, paternal involvement, and positive perceptions of giftedness as a source of confidence and opportunity. Findings suggest that, in the Korean gifted education context, achievement and social goals may be mutually reinforcing rather than competing. The study highlights the role of family support, competence beliefs, and culturally embedded social purpose in adaptive gifted development.

## 1. Introduction

Gifted adolescents are often portrayed as a homogeneous group driven primarily by academic achievement. Yet, mounting evidence suggests that gifted and high-ability students vary considerably in their psychosocial goal orientations. Person-centered and social-emotional research has identified distinct subgroups of gifted learners, including students with adaptive and maladaptive perfectionistic orientations, varied coping and adjustment patterns, and underachievement-related vulnerabilities that may not always visible through academic performance alone (Callahan et al., 2004; Dixon et al., 2004; Lo et al., 2025; Neihart, 1999). Related work on motivation further suggests that some gifted learners may become vulnerable to declining engagement when school environments provide insufficient challenge, belonging, or meaningful support (Hornstra et al., 2023; Ramos et al., 2025). Together, this literature suggests that gifted development cannot be understood solely through achievement outcomes; it also requires attention to the psychological, familial, and cultural factors that shape how gifted adolescents see themselves and their futures.

Life goals can be understood as cognitive representations of desired future outcomes that direct effort and identity development (Aarts & Elliot, 2012; Austin & Vancouver, 1996). They are especially important during adolescence, when young people begin to make sense of who they are and what futures they imagine for themselves. Prior research suggests that the types of goals students hold have significant implications for their mental health and life satisfaction. Prosocial life goals focused on relationships and community are associated with greater well-being (Kasser & Ryan, 1993; Lekes et al., 2010), whereas goals centered on wealth, fame, and status are linked to higher stress and maladaptive behaviors (Bradshaw et al., 2023; Niemiec et al., 2009). Life goals are therefore developmentally meaningful because they reflect both what adolescents hope to achieve and the values through which they organize their effort, relationships, and sense of purpose.

Adolescents’ life goals are not formed in isolation; they are shaped by family expectations and cultural values. In a study of multi-ethnic high-school seniors, Chang et al. (2006) found that life goals varied by ethnicity, gender, and generational status. Despite this insight, much of the goal-orientation literature has focused on Western student samples and typical learners rather than gifted populations. Comparatively little is known about the life goals that east Asian gifted adolescents hold related to career success, social contribution, relationships, or combinations of these aims. East Asian gifted students are an important group to study given their cultures’ emphasis on education and family. Confucian values in countries like Korea frame educational achievement as closely connected to familial duty and societal contribution (Kim & Park, 2000; Sun et al., 2023). Understanding Korean gifted students’ life goals can inform educators and parents about how these youths envision success and fulfillment beyond test scores.

The present study examines life-goal orientations among Korean gifted adolescents, focusing on two broad domains: achievement/career-oriented (AC) goals and social/relationship (SR) goals. We compare adolescents who strongly endorsed both domains (dual-goal group) with those who reported comparatively lower endorsement across both domains (low-goal group). We then examine whether these goal orientations are associated with differences in academic performance, family processes, and students’ perceived advantages of giftedness. The research questions are:

1. What patterns of achievement and social life goals are evident among Korean gifted high-school students?
2. Do students with dual-goal orientation differ from those with low-goal orientation in academic performance, family processes, and perceived advantages of giftedness?

### 1.1. Conceptual Framework

This study is guided by the view that life goals function as organizers of development. Heckhausen (1997) described life goals as organizing structures that orient young people toward major developmental tasks and guide decisions about education, work, relationships, family, and future roles. In adolescence, life goals are especially important because young people begin to imagine possible futures and connect present choices to longer-term aspirations (Nurmi, 1991). Goals help adolescents determine what is worth pursuing, where to invest effort, and how to interpret success, challenge, and opportunity (Austin & Vancouver, 1996). These future-oriented goals connect present behavior to anticipated futures and help adolescents make sense of academic demands, relationships, family expectations, and emerging identities.

Life goals are often described in terms of two broad categories (Bakan, 1966; Sheldon & Cooper, 2008): agentic (getting ahead) and communion (getting along). Agentic goals involve personal accomplishment such as striving for power or achievement, whereas communal goals involve connection with others, such as intimacy, affiliation, and altruism. In the context of adolescent aspirations, agentic goals may include earning high grades or becoming a leader in one’s field, while communal goals might include having a happy family or improving one’s community. In this study, we refer to these two orientations as achievement/career (AC) and social/relationship (SR) life goals. Students in the dual-goal group are those who reported relatively high endorsement of both AC and SR life goals. Students in the low-goal group are those who reported comparatively lower endorsement of both measured domains. Comparing these two groups allows us to examine academic performance, family processes, and perceived advantages of giftedness in relation to goal profiles.

Academic performance provides an important point of comparison because life goals are theorized to organize students’ effort, persistence, and educational decision-making over time (Alhadabi & Karpinski, 2020; Tang et al., 2021). If adolescents’ life goals shape what they value and what they are willing to work toward, differences in goal orientation may be reflected in academic outcomes. Family processes are also relevant to this framework, as parents play a central role in shaping adolescents’ future orientations by communicating which goals are valued, supported, and attainable (Grolnick et al., 1991; Marchant et al., 2001). Perceived advantages of giftedness are also examined because students’ subjective experiences of being gifted—such as confidence, recognition, good grades, stress, and access to opportunities—may shape what they believe their abilities make possible within their family, school, and broader social context (Gagné, 2004; Marsh & Craven, 2006; Subotnik et al., 2011).

### 1.2. Literature Review

#### 1.2.1. Multiple Goals and Gifted Adolescents

Research on student motivation increasingly suggests that students often pursue multiple goals at the same time rather than organizing their behavior around a single dominant aim. Earlier motivation research often contrasted mastery and performance goals, whereas later work showed that these goals can coexist and may be adaptive when students pursue both learning and achievement (Harackiewicz et al., 2002; Pintrich, 2000). A similar logic applies to achievement/career and social/relationship goals. These two domains should not be treated as competing priorities. Students may strive for academic success while also valuing relationships, belonging, family responsibility, and contribution to others.

Research on social goals supports this broader view of motivation. Wentzel (1991, 1993) found that adolescents’ academic success was linked not only to academic goals but also to social responsibility goals. Later studies similarly showed that social goals can support academic engagement, self-regulated learning, and positive attitudes toward cooperative learning when those goals are connected to belonging, contribution, and responsibility (Liem, 2016; Urdan & Maehr, 1995). At the same time, the effects of social goals depend on their content and context. Goals focused on contribution or belonging may support school engagement, whereas goals centered on popularity or peer approval may conflict with academic effort (Goagoses & Koglin, 2022; Lansu, 2023). The key question is therefore not whether students hold achievement or social goals, but how these goals are combined and made meaningful in students’ lives.

This perspective is especially relevant for gifted students. Gifted adolescents are often assumed to be highly achievement driven because they have been identified through academic or talent-based criteria and often receive recognition for exceptional performance. Giftedness, however, should not be equated with narrow achievement striving or self-focused ambition. Gifted education scholars have cautioned against stereotypes that frame gifted students as socially detached or motivated only by personal gain (Fiedler et al., 2002; Neihart, 1999). Prior research suggests that gifted adolescents may also express strong concern for purpose, contribution, and the use of their abilities for the benefit of others. For example, S. Y. Lee et al. (2020) found that academically gifted adolescents reported stronger prosocial goals and social engagement than non-gifted peers. Similarly, Çelik and Mertol (2018) found that gifted high school students often described long-term aims that connected achievement with societal contribution. These findings align with Renzulli’s (2012) view that gifted education should support both self-fulfillment and the development of creative contributors to society.

#### 1.2.2. Cultural and Family Context of Goal Development

The South Korean cultural context is crucial for understanding gifted adolescents’ life goals in this study. Like many other east Asian countries, South Korea is a collectivist society shaped by Confucian traditions that emphasize filial piety and deference to authority (Kim & Park, 2000). East Asian families and schools often place high value on academic excellence as a top priority, which is often linked to family honor and future socioeconomic stability. Markus and Kitayama (1991) noted that in collectivist cultures, achievement is frequently pursued as a way of fulfilling obligations to others, blurring the boundary between individual ambition and social responsibility. A personal achievement goal, such as entering a prestigious university, may therefore carry relational meanings, including making one’s family proud or becoming a responsible member of society. This merging of achievement and social duty is reflected in Tam’s (2016) study of high-achieving Korean adolescents, who described studying hard as a way to “repay parents” and bring honor to the family. Similarly, Jongho et al. (2014) found that gifted Korean youths expressed a relative balance between goals about personal gains, such as wealth and fame, and socially oriented goals. In interviews, students described social contribution as part of their adult role and expressed a responsibility to serve others in ways consistent with Confucian values of moral duty. In this cultural context, prosocial and achievement-oriented goals are not competing priorities; instead, they may become mutually reinforcing. Academic and career success can be understood as individual accomplishment, as a way to honor family support, and means of fulfilling broader social responsibility (S. Y. Lee et al., 2020).

#### 1.2.3. Goal Orientation Differences Among Gifted Adolescents

Giftedness does not imply a single developmental pathway or a shared outlook toward the future. Recent scholarship increasingly argues that gifted learners’ development must be understood not only in terms of academic performance or identification status, but also in terms of well-being and belonging (Rinn, 2024; Shaunessy-Dedrick & Suldo, 2025). This lens is especially important because highly selective educational settings can be double-edged: they may provide advanced opportunities and intellectual challenge, but they may also heighten stress and perfectionistic pressure (Helsper et al., 2025). If a gifted student’s self-worth becomes narrowly tied to competitive achievement, the student may become more vulnerable to anxiety or burnout (Grugan et al., 2025). By contrast, students whose future orientations include strong social and relational commitments may have additional resources for coping because they can ground their identity in multiple domains. This does not mean that social goals automatically protect gifted adolescents from stress; rather, it suggests that a dual-goal orientation may provide a more stable foundation for adaptive functioning in demanding academic settings. Recent work on gifted learners’ mental health similarly emphasizes flourishing as rooted not only in accomplishment, but also in relationships and civic engagement (Shaunessy-Dedrick & Suldo, 2025).

Within this broader developmental context, comparatively low goal endorsement may signal a weaker connection between gifted adolescents’ current academic experiences and their future-oriented aspirations. Some gifted students may experience declines in academic self-concept and motivation when they enter highly competitive environments (Hornstra et al., 2023), a phenomenon sometimes referred to as the big-fish–little-pond effect (Marsh, 1987). Others may experience academic work as externally imposed or disconnected from personal meaning. Family conditions also shape this process. Low parental support or excessive pressure may weaken students’ connection to school rather than strengthen motivation (Cho & Yoon, 2005). Under these conditions, gifted students may continue to perform at a high level while showing less clearly articulated achievement/career or social/relationship goals. This possibility is relevant to the present study because it examines whether gifted adolescents with stronger endorsement of both goal domains differ from those with comparatively lower endorsement across both domains.

## 2. Methods

This study used a cross-sectional comparative design to examine differences in academic performance, family processes, and perceived advantages of giftedness among Korean gifted adolescents with different levels of life-goal endorsement. Achievement/career and social/relationship goals were measured as continuous variables, consistent with research showing that students often pursue multiple goals simultaneously rather than holding mutually exclusive orientations (Dowson & McInerney, 2003; Pintrich, 2000). For analytic purposes, students with relatively high endorsement of both domains were grouped as dual-goal students, whereas students with comparatively lower endorsement of both domains were grouped as low-goal students. These labels were created to support exploratory group comparisons.

### 2.1. Participants

Participants were 83 gifted high-school students from various regions of South Korea. All students were identified as gifted in mathematics and science. Sixty-seven students attended specialized science high schools in Seoul, and sixteen students were enrolled in gifted programs at general high schools. South Korea’s specialized science high schools, governed by the national Ministry of Education, offer advanced and accelerated curricula for academically gifted students. Admission to these highly competitive magnet schools involves a rigorous three-step selection process, requiring candidates to rank in the top 2% citywide in science and mathematics, along with teacher recommendations and an oral examination (Seo, 2017). Gifted students from general high schools were selected on the basis of their performance on the Math (Cho & Han, 2004) and Science Creative Problem-Solving Test (Cho et al., 1997). These measures were used by the provincial offices of education to identify gifted students who scored in the top 3%. The sample represents a high-ability group, all of whom demonstrated exceptional academic potential. The initial sample of 138 students was reduced to 83 after 55 cases with incomplete survey data were excluded. Retained and excluded students did not differ significantly by gender, school setting, academic achievement, or perceived advantages of giftedness. Retained students reported significantly higher family support (*t* = 2.39, *p* = 0.021) and father involvement (*t* = 2.05, *p* = 0.045) than excluded students. The implications of this sample difference are addressed in the Limitations section.

Table 1 presents the demographic characteristics of the 83 students included in the final analytic sample. Parent education was generally high across the sample. About one-third of the students came from families with a monthly household income of USD 2720–USD 3390.

Because participants came from two school settings (science high schools and gifted programs in general high schools), we examined whether school type was associated with key study variables. School type was not related to students’ goal profiles (*χ*^2^(3, N = 83) = 3.16, *p* = 0.367). Students from science high schools were not more or less likely than students from general high-school gifted programs to fall into the dual-goal or low-goal group. School type also was not associated with AC goals, SR goals, academic achievement, family processes, or perceived advantages of giftedness. However, school type was associated with gender composition, *χ*^2^(1, N = 83) = 5.62, *p* = 0.018. Among science high-school students, 55 of 67 were male (82.1%) and 12 were female (17.9%). The general high-school gifted group was evenly divided, with 8 males and 8 females.

### 2.2. Measures

#### 2.2.1. Life Goal Orientations

Students’ life goals were assessed with a questionnaire developed for this study. Students rated the importance of 14 life goals on a 5-point Likert scale ranging from 1 (not at all important) to 5 (extremely important). The goals covered a broad range of aspirations related to the following: achievement and career aspirations, family and friendship, material wealth, recreation and leisure, and social contribution. Before examining the factor structure of the life-goal items, we assessed whether the item correlations were appropriate for factor analysis. The KMO value was 0.65, indicating acceptable though modest sampling adequacy for exploratory purposes (Kaiser, 1974; Tabachnick & Fidell, 2019). Bartlett’s test of sphericity was significant, *χ*^2^(21) = 163.79, *p* < 0.001, indicating that the items were sufficiently correlated to justify examining an underlying factor structure (Bartlett, 1954; Field, 2018). These results supported the use of exploratory factor analysis, while also suggesting that the factor solution should be interpreted cautiously.

To examine the underlying structure of the life-goal survey, we conducted principal components analysis with varimax rotation on the initial 14 goal items. Two clear factors emerged and accounted for 51.9% of the variance (see Table 2). The first factor reflected achievement/career (AC) goals, with items focused on personal accomplishments (e.g., being the best, attaining a high level of education, becoming a professional in a respected field). The second factor reflected social/relational (SR) goals, with items on relationships and societal contribution (e.g., maintaining close relationships with family and friends, helping others, striving for social equity). The final AC scale contained 3 items and had a Cronbach’s alpha of 0.54. The SR scale consisted of 4 items with a Cronbach’s alpha of 0.64. The remaining items did not load cleanly on either factor, and were not used in subsequent analyses. Because of the limited number of items and modest reliability estimates, the AC and SR scales were treated as exploratory indicators of goal orientation rather than fully validated measures.

For each student, responses to the retained items were summed separately to produce an AC score and an SR score. Students whose AC and SR scores were both above the sample medians were assigned to the dual-goal group, whereas students whose scores on both domains were below their respective medians were assigned to the low-goal group.

#### 2.2.2. Academic Performance

Grade point averages (GPAs) were obtained from participants’ school records across four school levels: early elementary school (Grades 1–3), upper elementary school (Grades 4–6), middle school (Grades 7–9), and high school (Grade 10). A cumulative GPA was calculated separately for each grade band, on a 7-point scale corresponding to letter grades from A to F. The four GPA measures were not averaged or combined into an overall cumulative academic performance score.

#### 2.2.3. Family Processes

We assessed several aspects of students’ family educational environments and parenting practices; using a 28-item survey originally developed by Campbell (1994) and adapted to the Korean context by Cho and Han (2004). Items were rated on a 5-point scale ranging from 1 (strongly disagree) to 5 (strongly agree) across four subscales. Responses were summed within each of four subscales. The four subscale scores were analyzed separately, and no overall family process score was calculated.

Parental support (5 items, α = 0.67): assessed emotional support and encouragement for schooling (e.g., “My parents encourage me when I have difficulties with my studies”).

Intellectual stimulation (8 items; α = 0.85): assessed the provision of cognitively rich environments (e.g., “There are many books and resources at home that help me learn new things”).

Educational cooperation between parents (8 items; α = 0.78): assessed the extent to which parents communicated about the child’s education (e.g., “My parents often talk together about my education”).

Paternal participation (7 items; α = 0.82) assessed fathers’ direct involvement in the child’s learning (e.g., “My father helps me with homework”).

#### 2.2.4. Advantages of Being Gifted

To assess students’ perceived advantages of being identified as gifted, we developed a five-item measure informed by prior research on gifted students’ perceptions of giftedness, gifted labeling, academic self-concept, and learning opportunities. The first domain, social recognition, assessed the extent to which students perceived gifted identification as leading others to recognize their superior ability. This domain reflects prior work showing that gifted students are aware of the social meaning of the gifted label and how it shapes others’ perceptions of them (Berlin, 2009; Tercan & Yildiz Biçakci, 2023). The second domain, academic confidence and ease, was informed by research on gifted students’ academic self-efficacy (Alabbasi et al., 2023; Marsh et al., 1995). The third domain, educational opportunities, reflected research showing that gifted students often describe gifted programming as providing distinctive academic opportunities and challenging learning experiences (Hertzog, 2003).

Students rated the extent to which they perceived certain benefits from being identified as gifted, on a 5-point scale ranging from 1 (strongly disagree) to 5 (strongly agree). The five items were as follows: (a) recognition by others of my superior ability, (b) high confidence in my capabilities, (c) earning good grades easily, (d) lower stress related to studying, and (e) opportunities for diverse experiences, such as special programs and competitions. For analysis, the five items were analyzed separately because they represented distinct perceived experiences. No cumulative perceived advantages score was calculated.

### 2.3. Data Collection

Surveys were administered in Korean, and all items were translated and back-translated for clarity. Before data collection, the school distributed and collected the consent and assent forms. Written informed consent was obtained from a parent or legal guardian, and students provided written assent. Students were assured that their responses were confidential and would not affect their grades or be shared with their teachers.

### 2.4. Data Analysis

To create goal-orientation groups, we conducted median splits on the AC and SR goal scores (median AC = 15.0, median SR = 12.0). Students scoring above the median on a given scale were classified as high on that goal; those scoring at or below the median were classified as low. This yielded four groups: (1) high achievement/high social–relational goals (dual-goal orientation, n = 37), (2) high achievement/low social–relational goals (n = 11), (3) low achievement/high social–relational goals (n = 10), and (4) low achievement/low social–relational goals (low-goal orientation, n = 25). Because the single-domain groups were small, subsequent analyses focused on comparisons between the dual-goal (DG) and low-goal (LG) groups. These two groups were selected because they represented the clearest contrast across both goal dimensions.

Descriptive statistics were computed separately for the dual-goal (DG) and low-goal (LG) groups. Independent-samples *t*-tests were then conducted to compare the two groups in three areas: academic performance, family processes, and the five perceived advantages of being gifted. As a supplementary analysis, separate bivariate logistic regressions were conducted to express each association in terms of the odds of dual-goal (DG) group membership. The *t*-tests indicated whether the DG and LG groups differed significantly on academic performance, family processes, and perceived advantages of being gifted. The logistic regressions produced odds ratios showing the direction and strength of each variable’s association with DG membership. Each predictor was tested in a separate model because the subgroup sizes were modest and a multivariable model would have increased the risk of overfitting.

## 3. Results

### 3.1. Descriptive Pattern of Goal Profiles

Students in the DG group reported higher mean scores on both goal subscales than students in the LG group (see Table 3). The DG group had higher SR goal scores (*M* = 4.41, *SD* = 0.35) than the LG group (*M* = 3.25, *SD* = 0.24). The DG group also reported higher AC goal scores (*M* = 4.64, *SD* = 0.27) than the LG group (*M* = 3.44, *SD* = 0.33).

### 3.2. Comparison of Dual-Goal and Low-Goal Groups

Table 4 presents the descriptive statistics and independent-samples *t*-tests results comparing the DG (n = 37) and LG (n = 25) groups on academic GPA, survey scores on family processes and perceived advantages of being gifted. At the individual schooling levels, DG students reported significantly higher middle-school GPA than LG students (*t*(60) = 2.36, *p* < 0.05). The effect size was moderate (Cohen’s *d* = 0.64). Differences in lower-elementary, upper-elementary, and high-school academic performance were not statistically significant.

Group differences were statistically significant for all four subscales of family processes, and the effect sizes were large, ranging from 0.88 to 1.15. Students in the DG group reported more support from their parents, *t*(60) = 2.99, *p* < 0.01, and greater intellectual stimulation at home, *t*(60) = 2.47, *p* < 0.05. They also reported higher educational cooperation between parents, *t*(60) = 3.04, *p* < 0.01, and greater paternal involvement in their education than their LG peers, *t*(60) = 3.22, *p* < 0.01.

The DG students also reported experiencing significantly greater benefits associated with being gifted than the LG group on four out of five indicators: (1) receive positive recognition from others for their abilities, (2) have increased confidence in their capabilities, (3) obtain good grades more easily, and (4) enjoy diverse opportunities as a result of their gifted status. The effect sizes were large, ranging from 0.97 to 1.51. On the one item “I have less stress for study,” there was no significant difference.

### 3.3. Logistic Regression Models for Associations with Dual-Goal Group Membership

Results from the supplementary bivariate logistic regressions are reported in Table 5. Among the individual academic performance variables, only middle-school GPA was significant, OR = 2.86, *p* = 0.021. Each family-process subscale was also significant, including parental support, OR = 1.48, *p* = 0.016; intellectual stimulation, OR = 1.20, *p* = 0.031; educational cooperation between parents, OR = 1.27, *p* = 0.013; and paternal participation, OR = 1.24, *p* = 0.010.

Four of the five perceived advantages of giftedness were significantly associated with dual-goal membership. Higher perceived praise or recognition, OR = 3.46, *p* = 0.003; increased confidence, OR = 14.97, *p* < 0.001; better grades, OR = 3.45, *p* = 0.003; and diverse opportunities, OR = 4.11, *p* = 0.001, were associated with greater odds of dual-goal membership. Perceived reduction in study-related stress was not significantly associated with DG membership.

## 4. Discussion

Guided by the view that life goals function as organizers of development, this study compared gifted Korean adolescents who reported relatively high endorsement of both achievement/career and social/relationship goals with those who reported comparatively lower endorsement of both domains. Across analyses, students in the dual-goal group reported stronger middle-school academic performance, more supportive and intellectually stimulating family processes, greater paternal involvement, and more positive perceived advantages of giftedness than students in the low-goal group. These results challenge the notion that gifted students are a homogeneous group driven only by achievement. On the contrary, a substantial portion of these high-achieving adolescents valued interpersonal relationships and civic responsibility even within a highly competitive academic environment. For these students, doing well and doing good appeared to be mutually reinforcing rather than competing priorities.

### 4.1. Dual-Goal Orientation as a Developmental Strength

The dual-goal pattern is consistent with a multiple-goals perspective, which suggests that achievement goals and social goals can operate together in students’ academic development. Students do not necessarily choose between striving for academic success and valuing relationships or social contribution. These goals may reinforce one another when students see achievement as connected to family responsibility, future contribution, and meaningful participation in society. This interpretation is especially relevant in the Korean context, where educational achievement is often tied to family expectations, social mobility, and collective responsibility.

From this perspective, the stronger academic and developmental profile of dual-goal students may reflect more than high achievement motivation alone. These students appeared to experience achievement as connected to a broader set of meanings. Their academic and career aspirations were accompanied by strong endorsement of social and relationship goals, suggesting that success may have been understood not only as individual advancement but also as a way to maintain valued relationships and contribute to others. This finding aligns with prior research showing that social goals can support academic motivation when they are compatible with students’ educational values and social contexts (Horst et al., 2007; Urdan & Maehr, 1995; Wentzel & Wigfield, 1998).

The dual-goal orientation observed in this study also mirrors the dual cultural narrative in Korea described by Jongho et al. (2014). Korea’s educational system strongly rewards achievement. For many students, admission to an elite college like Seoul National University represents the ultimate academic aspiration, and many gifted students feel the weight of that expectation (Bae & Choi, 2024; Choi & Nieminen, 2013). At the same time, Korean cultural values emphasize humility, family duty, and using one’s abilities for the greater good (Jongho et al., 2014). Gifted Korean adolescents grow up in a context in which academic success is often interpreted through family and social meanings (S. Y. Lee et al., 2020; Tam, 2016). Doing well in school may be understood as honoring parents, fulfilling family expectations, and preparing oneself to contribute to society. Achievement goals may carry relational significance, and social goals may strengthen the meaning of academic striving. The dual-goal students in this study appear to embody this connection between individual accomplishment and social responsibility. These findings suggest that, for at least some Korean gifted adolescents, high achievement is not sustained by competitive ambition alone. Rather, social goals appear to coexist with—and possibly amplify—achievement aspirations.

### 4.2. Lower Goal Orientation Among Gifted Adolescents

Students in the low-goal group should not be understood as lacking goals, motivation, or future aspirations. Rather, they reported comparatively lower endorsement of both achievement/career and social/relationship goals within this gifted sample. This distinction is important because these students were still identified as gifted and continued to endorse some future-oriented aspirations. For example, becoming an expert or professional in one’s field remained meaningful to many students in this group. The concern, therefore, is not the absence of goals but the weaker expression of future-oriented goals across the two measured domains.

This interpretation is particularly relevant in the Korean cultural context. Korean gifted adolescents develop in a society where academic achievement is often tied to family expectations, social mobility, and collective responsibility (Kim & Park, 2000; Markus & Kitayama, 1991; Sun et al., 2023). For dual-goal students, achievement and social responsibility appeared to reinforce one another. For low-goal students, these connections may have been less salient. Their lower endorsement of both goal domains may therefore signal a less developed link between academic ability and the broader meanings that can sustain effort in demanding educational environments. Without strong achievement/career goals, the low-goal gifted adolescents may be less motivated to invest consistent effort in academics, particularly when coursework becomes challenging. And without strong social/relationship goals, they may lack a sense of connection or obligation that could anchor them in school or family communities.

Giftedness alone does not guarantee a strong sense of direction, purpose, or sustained engagement. Motivation research suggests that students’ engagement depends not only on ability, but also on the value and meaning they attach to academic tasks and future goals (Eccles & Wigfield, 2002; Pintrich, 2000). Academic vulnerability may not always appear first as low performance. Gifted students can continue to perform adequately while motivation, task value, engagement, or sense of purpose begins to weaken (Reis & McCoach, 2000; Snyder & Linnenbrink-Garcia, 2013). The present study does not show that low-goal students were underachieving. However, comparatively lower endorsement of both achievement/career and social/relationship goals may be worth monitoring because it could reflect a less robust motivational foundation for sustained talent development. Future longitudinal research is needed to determine whether lower endorsement across both goal domains is associated with later decline in achievement.

### 4.3. Family Processes and Paternal Involvement

The family findings are consistent with research showing that family influence is a major environmental factor in shaping students’ motivation, self-regulation, and goal pursuit (Collins & Steinberg, 2008). When students experience encouragement and intellectual stimulation at home, they may be more likely to view academic striving as meaningful and attainable. When parents communicate about education and provide coordinated support, students may receive clearer messages about the value of effort, learning, and long-term planning. Prior research has also shown that children’s perceptions of parental support and involvement are associated with motivational resources and school achievement (Grolnick et al., 1991). These findings also align with talent development models that emphasize the interaction of ability, learning opportunities, and psychosocial support from both educational institutions and families over time (Gagné, 2004; Subotnik et al., 2011). In the present study, these family processes were linked to a goal profile that combined achievement/career aspirations with social/relationship commitments.

Father involvement is particularly noteworthy in the Korean context. Historically, Korean fathers were expected primarily to serve as family breadwinners, while mothers have often taken more direct responsibility for children’s daily care and educational management (Roh & Yang, 2013). When fathers are visibly involved in educational decision-making and academic support, their participation may carry symbolic importance. It may signal that the child’s development is a shared family priority and that academic effort is valued within the broader family system (Park, 2018). The higher level of paternal involvement found in the dual-goal group mirrors recent research findings that show that Korean fatherhood is becoming more diverse, with fathers varying in accessibility, caregiving, and direct involvement with children. Ko et al. (2022) identified patterns of Korean father involvement, including a highly involved group characterized by active childcare, accessibility, and socialization behaviors. A high level of paternal participation may help reinforce both achievement/career goals and social/relationship goals. This interpretation is consistent with research on young Korean gifted children showing that high parental support and involvement were associated with stronger learning motivation (Otani, 2019), whereas low father involvement was linked to more psychosocial difficulties (Cho & Yoon, 2005). However, greater paternal involvement is not necessarily beneficial in all forms (W. K. Lee & Kim, 2024). In highly competitive educational contexts, parental involvement may become harmful when students experience it as pressure or control (Xu et al., 2020; Zhang et al., 2023). What appears most adaptive in the present study is a family pattern that combines emotional support, intellectual stimulation, parental cooperation, and paternal participation. These conditions may help gifted adolescents pursue achievement while grounding that achievement in relational belonging and broader social purpose.

### 4.4. Perceived Advantages of Giftedness

Perceived advantages of giftedness were also closely connected to dual-goal membership. Compared with students in the low-goal group, students in the dual-goal group were more likely to report that being gifted brought praise or recognition, increased confidence, better grades, and access to diverse opportunities. These findings suggest that gifted identification may have functioned as an enabling identity for these students, shaped by both external affirmation and internal self-belief. Among the perceived advantages, confidence showed the strongest association with dual-goal membership, suggesting that students who believe in their capabilities may be more willing to pursue ambitious academic and career goals while also imagining socially meaningful uses of their abilities. This interpretation is consistent with research showing that competence beliefs are central to students’ motivation, choices, and persistence (Eccles & Wigfield, 2002). The findings also point to the importance of recognition and opportunity. When gifted students receive positive recognition from others and access to enriched experiences, they may come to view their abilities as valuable resources that can support both personal achievement and contribution to others.

The two groups did not differ significantly in perceived stress. This suggests that the advantages of giftedness did not necessarily make academic life feel less demanding. In a competitive educational context, giftedness may bring benefits and pressures at the same time. What distinguished the dual-goal students was the presence of more positive meanings attached to their giftedness, rather than the absence of stress.

### 4.5. Academic Performance and Goal Orientation

The clearest academic difference appeared in middle school, where dual-goal students reported significantly higher GPA than low-goal students. This timing is meaningful because middle school is often a period when academic work becomes more demanding and students begin to connect present performance with future educational pathways. The middle school finding suggests that differences in goal orientation may become more visible when academic expectations and future planning become more salient.

By high school, the dual-goal group still reported a higher mean GPA than the low-goal group, but the difference did not reach statistical significance. This pattern should be interpreted cautiously. The continued mean difference suggests that the academic gap observed in middle school did not disappear entirely, but high-school GPA is more difficult to interpret because students were then enrolled in different types of educational settings, including science high schools and gifted programs in general high schools. Although preliminary analyses indicated that school type was not significantly associated with goal-profile membership, high-school grades may still reflect differences in curriculum and grading practices. Therefore, the high-school results suggest a possible pattern, but do not provide conclusive evidence of a distinct difference between the groups. Overall, the academic results indicate that the association between dual-goal orientation and academic performance was most evident during middle school, while any continuation of this pattern into high school remains less certain.

## 5. Implications

The findings have several implications for educators, parents, and gifted education programs. The results suggest that placement in gifted programs may need to be accompanied by intentional support for students’ goal development. Students in the dual-goal group showed a more favorable academic and developmental profile than students with comparatively lower endorsement of both achievement/career and social/relationship goals. This pattern suggests that gifted students may benefit when they are supported in connecting academic effort to meaningful future aims, including career aspirations, family relationships, and contribution to others. Teachers, counselors, and program coordinators can help students explore the personal and social meanings of achievement through mentoring, career exploration, service learning, research projects, and guided reflection. These practices may help students connect academic work to longer-term purposes, including future careers, family responsibilities, community participation, and social contribution.

The findings also challenge narrow stereotypes that portray Asian students as focused on academic achievement to the exclusion of other aspects of life. In this study, students with the more favorable academic and developmental profile were not characterized by achievement goals alone; they also valued social relationships and contribution. This pattern challenges achievement-only narratives that often dominate high-stakes educational systems. Messages that emphasize only academic success are not conducive to academic and psychosocial development. In fact, helping gifted adolescents connect academic effort with relational belonging may provide a more adaptive foundation for success. For Asian families and elite schools in particular, the results suggest that encouraging students to value relationships and community is not a distraction from academic excellence; it may be part of what sustains it.

The findings further highlight the importance of family processes in gifted adolescents’ goal development. Dual-goal students reported more supportive family environments, including parental encouragement, intellectual stimulation, parental communication about education, and father involvement. These findings suggest that families may play an important role in helping gifted students understand achievement as meaningful and attainable. Parent education programs in gifted education should therefore move beyond advice about academic performance alone. They should also help parents support students’ confidence, autonomy, sense of purpose, and relational well-being. In the Korean context, where educational success is often closely tied to family expectations, this balanced form of support may be especially important. Families can encourage high achievement while also helping students connect their abilities to relationships, responsibility, and contribution.

Finally, the non-significant finding for study-related stress has an important practical implication. Dual-goal students did not report that giftedness reduced their study-related stress. This suggests that even students with strong goal orientations and positive perceptions of giftedness may continue to experience academic pressure. Gifted programs should therefore avoid assuming that high-performing or highly motivated students are doing well simply because they appear successful. Support for gifted adolescents should include attention to stress, identity development, peer relationships, and students’ sense of purpose. A balanced approach to gifted education should support excellence while also helping students develop the relational and social meanings that can make achievement more sustainable.

## 6. Limitations and Future Research

The study is cross-sectional and based on retrospective self-report, so causal claims cannot be made. Longitudinal research that follows children from elementary through high school would allow for more precise tracking of goal formation and change over time. The creation of goal-profile groups through median splits is another limitation. This approach reduced variation in the original continuous goal scores and may have placed students into different groups based on relatively small score differences. In addition, the present analysis focused on the dual-goal and low-goal groups because these groups provided the clearest contrast between students with relatively higher and lower endorsement of both goal domains. However, this analytic choice excluded students who strongly endorsed only achievement/career goals or only social/relationship goals. Future studies should use latent profile analysis or cluster analysis on larger samples to identify goal profiles and examine all possible goal configurations more fully.

The small sample size and missing data also limit the interpretation of the findings. A substantial portion of the sample was excluded from the data analysis due to incomplete surveys. Students who completed all the surveys and remained in the analytical sample reported higher levels of family support than students who were excluded. Consequently, the analytic sample may overrepresent students with stronger family support. Future studies should use more rigorous data collection protocols to minimize missing data.

The survey instruments also require further development. The achievement/career and social/relationship goal scales were based on a small number of items and had modest internal consistency. Therefore, they should be interpreted as exploratory indicators of goal orientation rather than fully validated measures. Similarly, the survey on the perceived advantages of giftedness was developed for this study and requires further validation.

Finally, because the sample consisted only of Korean gifted adolescents, generalization of this study’s findings to other gifted populations should be made cautiously. In interpreting our results, we noted that social goals here likely complement academic goals within the context of Korean collectivist culture. In more individualistic cultures, social goals may take different forms or interact differently with achievement goals. Future research should examine culture as a potential moderator.

## Figures and Tables

**Table 1:** Demographic characteristics of the participants.

Characteristic		Science High School (n = 67)	General High School (n = 16)	Total (n = 83)
Gender	Male	55 (82.1%)	8 (50.0%)	63 (75.9%)
	Female	12 (17.9%)	8 (50.0%)	20 (24.1%)
Father’s Highest Education	Elementary School	1 (1.6%)	0 (0.0%)	1 (1.3%)
	High School	10 (16.4%)	4 (25.0%)	14 (18.2%)
	College	23 (37.7%)	10 (62.5%)	33 (42.9%)
	Graduate School	27 (44.3%)	2 (12.5%)	29 (37.7%)
	missing response	6	0	6
Mother’s Highest Education	Elementary School	1 (1.6%)	0 (0.0%)	1 (1.3%)
	Middle School	1 (1.6%)	0 (0.0%)	1 (1.3%)
	High School	14 (23.0%)	8 (50.0%)	22 (28.6%)
	College	39 (63.9%)	7 (43.8%)	46 (59.7%)
	Graduate School	6 (9.8%)	1 (6.2%)	7 (9.1%)
	missing response	6	0	6
Household Monthly Income	USD 680–USD 2030	9 (14.5%)	4 (25.0%)	13 (16.7%)
	USD 2040–USD 2710	16 (25.8%)	2 (12.5%)	18 (23.1%)
	USD 2720– USD 3390	21 (33.9%)	5 (31.2%)	26 (33.3%)
	USD 3400–USD 4750	12 (19.4%)	3 (18.8%)	15 (19.2%)
	USD 4750–USD 6780	3 (4.8%)	1 (6.2%)	4 (5.1%)
	USD 6790 or more	1 (1.6%)	0 (0.0%)	1 (1.3%)
	missing response	5	1	6

Note. Household monthly income was originally reported in Korean won. U.S. dollar equivalents are approximate based on an exchange rate of about KRW 10,000 = USD 6.79.

**Table 2:** Factor loadings of the life-goal survey items across the two goal dimensions.

Subscale	Item	Social/Relational Goals	Achievement/Career Goals
Social/Relational (SR) goals	Having good friendships	0.60	0.09
	Serving or helping society	0.80	0.14
	Maintaining close family relationships	0.69	0.17
	Contributing to reducing social inequality	0.64	0.07
Achievement/Career (AC) goals	Becoming one of the best in the world	0.11	0.52
	Becoming an expert/professional in my field	0.14	0.81
	Obtaining a good education	0.12	0.85

**Table 3:** Descriptive statistics of life-goal survey items for dual- and low-goal orientation groups.

Subscale	Item	Low-Goal *M* (*SD*)	Dual-Goal *M* (*SD*)
Social/Relational (SR) goals	Having good friendships	3.88 (0.60)	4.78 (0.48)
	Serving or helping society	2.84 (0.47)	4.32 (0.58)
	Maintaining close family relationships	3.60 (0.82)	4.70 (0.46)
	Contributing to reducing social inequality	2.68 (0.48)	3.84 (1.01)
Achievement/Career (AC) goals	Becoming one of the best in the world	2.60 (0.71)	4.22 (0.71)
	Becoming an expert/professional in my field	4.24 (0.66)	5.00 (0.00)
	Obtaining a good education	3.48 (0.59)	4.70 (0.57)

**Table 4:** Group comparisons between low-goal and dual-goal students.

	Variables	LG Group		DG Group		*t*	95% CI	Cohen’s *d*
		*M*	*SD*	*M*	*SD*			
Academic performance	Lower elementary	5.80	1.08	5.94	1.51	0.41	[−0.52, 0.81]	0.11
	Upper elementary	6.16	0.85	6.35	0.95	0.81	[−0.27, 0.65]	0.21
	Middle school	6.32	0.69	6.70	0.52	2.36 *	[0.05, 0.71]	0.64
	High school	4.44	1.78	5.16	1.59	1.67	[−0.17, 1.61]	0.43
Family processes	Parental support	18.79	2.58	21.72	2.89	2.99 **	[0.96, 4.92]	1.07
	Intellectual stimulation	28.71	5.22	33.06	4.71	2.47 *	[0.68, 8.00]	0.88
	Educational cooperation between parents	29.50	4.55	34.39	4.49	3.04 **	[1.59, 8.19]	1.08
	Paternal participation	25.86	5.53	32.06	5.31	3.22 **	[2.23, 10.17]	1.15
Perceived advantages of being gifted	Recognition	3.33	1.13	4.35	0.89	3.93 ***	[0.34, 1.26]	0.97
	Increasing confidence	3.58	1.02	4.68	0.63	5.20 ***	[0.54, 1.21]	1.51
	Good grades	3.33	1.13	4.24	0.72	3.84 ***	[0.32, 1.21]	0.96
	Low stress	2.79	1.29	3.38	1.26	1.77	[−0.17, 1.11]	0.38
	Diverse opportunity	3.46	1.18	4.68	0.88	4.60 ***	[0.51, 1.44]	1.22

Note. * *p* < 0.05, ** *p* < 0.01, *** *p* < 0.001.

**Table 5:** Bivariate logistic regression predicting dual-goal membership.

	Predictor	N	B	SE	Wald	OR	95% CI for OR
Academic performance	Lower elementary	61	0.08	0.19	0.17	1.08	[0.74, 1.59]
	Upper elementary	62	0.23	0.29	0.66	1.26	[0.72, 2.22]
	Middle school	62	1.05	0.46	5.29	2.86 *	[1.17, 7.02]
	High school	62	0.26	0.16	2.64	1.29	[0.95, 1.76]
Family processes	Parental support	32	0.40	0.16	5.84	1.48 *	[1.08, 2.05]
	Intellectual stimulation	32	0.18	0.08	4.67	1.20 *	[1.02, 1.41]
	Educational cooperation between parents	32	0.24	0.10	6.14	1.27 *	[1.05, 1.54]
	Paternal participation	32	0.21	0.08	6.57	1.24 *	[1.05, 1.45]
Perceived advantages of being gifted	Recognition	59	1.24	0.42	8.59	3.46 **	[1.51, 7.93]
	Increasing confidence	59	2.71	0.76	12.52	14.97 ***	[3.34, 66.98]
	Good grades	60	1.24	0.42	8.78	3.45 **	[1.52, 7.83]
	Low stress	60	0.31	0.22	2.01	1.37	[0.89, 2.12]
	Diverse opportunities	59	1.41	0.41	11.84	4.11 ***	[1.84, 9.20]

Note. * *p* < 0.05, ** *p* < 0.01, *** *p* < 0.001.

## Data Availability

The data presented in this study are not available due to ethical and privacy restrictions.
